# Buffalo Academy of Medicine

**Published:** 1901-01

**Authors:** Frederick H. Millener


### Society Proceedings.

### BUFFALO ACADEMY OF MEDICINE.

*Section on Surgery, October 2, 1900.*

REPORTED BY FREDERICK H. MILLENER, M. D., Secretary.

THE second regular meeting of the Surgical section, for the season of 1900, was held on October 2d, thirty-two being present.

The minutes of the preceding meeting were read and approved.

The essayists of the evening were SYDNEY A. DUNHAM and HERMAN E. HAYD.

Dr. SYDNEY A. DUNHAM delivered a paper on

### TRAUMATIC INSANITY.

The patient was born in 1858, in Germany. The father was killed by accident. His mother died at fifty-six years of age. His father had a sister insane. The patient was injured at the age of twenty-four. He was knocked down and remained unconscious for two hours. Next day he found a large sore spot on top of his head. He had been unable to work for the last two years, and has been attended by Dr. H. B. Brownell. On examining his head, a depression on the skull was found at the point of injury. He was sent to the Riverside Hospital and a bone removed of about one inch in diameter. He was there ten days and went home feeling well. He said his head felt lighter. He was only home a few days when, while sawing wood, he hit his head against a beam, and this was followed by delirium for two days. He rested in bed quietly and in a few days was apparently all right. Previous to the operation any sharp noises startled him. He would lose himself at church and could not remember anything stated at church. The children disturbed him at home and he was unable to saw wood straight. He was previously an experienced man with the saw. He moved his house back into the yard instead of towards the front, as he intended. He could not even take a glass of beer without losing himself.

In discussing the paper, Dr. H. B. BROWNELL stated: I have only a few words to say about this case of Mr. Abel's. I was called and found Mr. Abel in a greatly exhausted condition. He had come home a trifle late from his work, and had evidently stopped and taken a good deal of beer, as he appeared to me to be suffering from alcoholism. I gave him some bromide and had him go to bed. He was fairly comfortable next morning. I saw him occasionally during the next four weeks. I knew him at the time he had this accident a number of years ago and did not think that that accounted for any mental disturbance he might have. He always insisted upon having beer, and when not able to get it himself would send out for it, until I told him it was impossible for him to get well if he took it. His wife pestered him a good deal, and at the time I thought that had something to do with it. He had some delusions that he was being persecuted or pursued by some one, and I thought the best place for him was an asylum. He complained at different times of pains in his head, and, as I remember, his pain was more to the base and over the side of the injury, although I may be a little mistaken about that, as it never occurred to me at that time that his trouble might be due to his accident. Since this man has been trephined he has been quite well; therefore, I am forced to the conclusion that the accident was the cause of his trouble.

Dr. Herman E. Hayd presented a paper on

### TREATMENT OF CHOLELITHIASIS.

He divided the treatment into three divisions: (1) the prevention of cholelithic condition; (2) the relief of an attack of gallstone colic; (3) the emptying of the gall-bladder and ducts of their stones. First is met by encouraging outdoor, active life, moderation in food and alcoholic drinks, massage and hot baths daily and free bowel movement, the lessening of any constriction and abdominal viscera by corsets, tight waists, or belts and the like. It is also stated on good authority that the very free use of starches and non-nitrogenous foods encourage the tendency to the formation of gallstones by lessening the amount of bile acids in the bile, and for that reason a meat diet has been suggested. During an attack of colic, the pain must be met by hypodermic injections of morphia or even inhalations of chloroform; the application of a large hot poultice of linseed meal to the abdomen over the liver areas; a prolonged hot bath, which sometimes relieves the muscular and nervous system to such an extent as to favor the exit of the stone, and if shock and collapse be profound, such remedies as would be naturally indicated.

When stones have forced an exit in the gall-bladder or ducts there is no medicine which causes their absorption or liquefaction. It is perhaps true, however, that a course at Carlsbad or the regular use of aperient waters might help their discharge into the bowel, first by relieving associated congestion and catarrh and, perhaps, by helping on the bile current. It is in this way that an explanation can be found for the apparent good of the olive oil treatment, because there can be no doubt, in my mind, that relief has come from its prolonged administration. Not by a saponifying and dissolving stones, but by antagonising and soothing catarrhal processes. So, with patients who go to the various spas and springs in Germany, after a few days' sojourn, they begin to pass stones, and this fact can only be explained upon the supposition that the onward flow of the bile is stimulated and the catarrhal ducts are rendered less sensitive and more patulous. However, when the gall-bladder, or the ducts, is the seat of the stones, which are not being gotten rid of, and are causing much pain and irritation, a surgical operation is indicated.

Dr. Dunham's paper was discussed by Drs. Frost, Matzinger, Fell and Eugene A. Smith.

Dr. Frost described several cases which had come under his observation at the State Hospital, and reported one case which had not been particularly benefited by operation.

Dr. Matzinger called attention to a case of traumatic insanity, the wife of one of the employees of the hospital. She was suffering from chronic mania following confinement. She was relieved after an operation.

Dr. Fell described a case which had come under his observation, where a patient had fallen out of a street car on Elmwood avenue and North street, and considered that there might possibly be a bone pressing on the brain.

Dr. Eugene A. Smith described a man at the General Hospital, who was struck on the side of the head while passing a bridge on a canal boat. He stated that if ever anybody was generously trephined, he was. A greater portion of the parietal and a considerable part of the frontal bone was removed. He was insane and is in the hospital now.

Dr. Hayd's paper was discussed by Drs. Thoma, Eugene A. Smith and Colton.

Dr. Eugene A. Smith said: I find both papers interesting. I do not care to go over the ground that Dr. Hayd went over as to discussion, although I wish to add that infection is usually the cause of formation of stones in the gall-bladder.

Dr. COLTON described a case which he had been called to and had operated upon.

Dr. THOMA stated that gallstone is very common. There are many times when the patient will not be operated upon, and also that he frequently removed them by giving olive oil. He believed, however, that cases of undoubted gallstone ought to be operated upon.

The meeting adjourned at 11 p. m.

---

*Section on Medicine, November 13, 1900.*

REPORTED BY JULIUS ULLMAN, M. D., Secretary.

The second regular meeting of the Section on Medicine of the Buffalo Academy of Medicine, for the season, was held in the Library building, Tuesday evening, November 13, 1900, the chairman, Dr. ELI H. LONG, presiding.

Dr. WILLIAM C. KRAUSS read a report of a case of

ASTHENIC BULBAR PARALYSIS

occurring in a man 55 years of age, who had up to a year ago enjoyed the best of health. Habits, with exception of a period of five years, when he was engaged in politics, good. About a year ago he began to notice difficulty in swallowing, occurring especially when he was fatigued or under nervous excitement. Then gradually the speech became labored and heavy; especially difficult was it to pronounce the labials. The lips would draw up so that he could not grasp the cup in taking food. With these symptoms there followed a paretic condition of the whole muscular system. The face muscles were slow, responded tardily, the left eyelid was markedly paretic, lagophthalmos was present at night, could not masticate solid food, jaw felt heavy, difficulty in walking, hands and arms were perceptibly weakened. Choking spells set in so that his life was despaired of, and he was given the blandest form of liquid foods.

Examination revealed a man of strong physique, large skeleton, 5 feet 7 inches in height, former weight 236 pounds, present weight 140 pounds; face stolid, expressionless, with ptosis of left eyelid, atony of facial muscles, thin retracted lips, tongue does not extend beyond the inner margin of the lips and not atrophied, palate immovable but hanging in the middle line, inability to pucker the lips or inflate the buccal cavity; throat shows nothing abnormal, except some retraction of the left vocal chord after exercise; sensation of face and body unimpaired; muscles soft and flabby; tendon reflexes somewhat diminished; no disturbances of the rectal or vesical reflexes; urine normal. Pulse at times weak and lowered.

Treatment consists of liquid diet, strychnine, galvanism to the head and neck and general faradisation. Improvement has apparently taken place, as he is able to speak and swallow much better, is stronger on his feet and has more control over his hands. This improvement is doubtless only temporary and prognosis is considered unfavorable.

Dr. Fridolin M. Thoma showed a boy eight years of age with a history that he was slow to walk and that he slid about until two years of age. There was also a rapid closure of the fontanelles. There is an absence of knee-jerks, a weakness of muscular power in the lower extremities and a pseudo-hypertrophy of the muscles of the calf of the leg. The boy rises from the ground in the characteristic manner as described by Gowers, and gives a very interesting symptom complex of pseudo-hypertrophic muscular paralysis.

A REVIEW OF THE MODERN MEDICAL AND SURGICAL TREATMENT OF EPILEPSY, was the title of a paper read by Dr. L. Pierce Clark, physician at the Craig Colony of Epileptics at Sonyea, N. Y. The substance of Dr. Clark's instructive paper may be appropriately given in his general conclusions. He arranges the present status of trephining as follows: 1. Idiopathic epileptics with typical grand mal seizures should never be trephined. 2. Idiopathics, in whom seizures are of the Jacksonian type, should only be trephined when infantile palsies can be excluded and when the family and personal degeneracy is at a minimum; even then but a fraction of 1 per cent. of this class recover. If these cases are operated upon, a very thorough removal of the epileptogenic area should be made and we should expect a more or less permanent paresis, according to the extent and the thoroughness of the cortical removed. 3. Traumatic epileptics should be trephined only when the injury is definitely proven and stand in direct causal relation, has existed not more than two years, and in the absence of a neurotic family history. The earlier trephining is resorted to after convulsions begin, the better the prognosis. The remnants of palsy lesions in infancy must be carefully excluded. If these rules are followed, many less so-called traumatic cases will be trephined, but the result will far exceed 4 per cent. of recoveries. All epileptics trephined from whatever cause must be given post-operative bromide treatment for years.

The present status of medical treatment is as follows: 1. By a combination of diet, regular occupation and personal hygiene, the bromides gain for us the best results in treating idiopathic epilepsy. 2. The bromides, singly or combined, still hold a very important place in epileptic treatment and still remain our chief sedative for the epileptic state: in the young epileptic, to secure a possible entire suppression of attacks and ultimate cure of the disease; in the adult, an amelioration of the frequent paroxysms. 3. The bromides, to be effective in chronic and long standing cases, must be given in large daily doses to suppress convulsions—from 300 to 400 grains, if necessary. They should be given gradually to find the sedative level, to which level it is the physician's chief duty to maintain them with physical and mental comfort to the patient. 4. Hot and cold baths, high enemas, alimentary antisepsis and massage are absolutely essential to successful bromide medication. 5. Bromine is a worthy substitute to the bromides in many cases when the latter are contraindicated or cannot be given in high dosage. 6. Salt starvation or semi-salt starvation is a great adjuvant to the bromide treatment and should be thoroughly tried in all cases where bromides are apparently contraindicated before the salt is discarded.

PARESIS AND CEREBRAL SYPHILIS.

Dr. ARTHUR W. HURD, superintendent of the Buffalo State Hospital, read an article with the above-named title. The type of disease which has been called pseudo-paresis and the resemblance between paresis and cerebral syphilis, including the points of differential diagnosis, as well as the points of marked resemblance, were first considered.

He described the origin, onset and duration of the two conditions, raising the question of the possibility of an as yet unrecognised toxin of syphilis acting in cases of paresis, which appear five to twenty years after infection, as compared with the specific virus as ordinarily seen. He considered the importance of differential diagnosis and reviewed briefly the work of Dr. Mott, pathologist of the London County Asylums, as to the pathological process which goes on in paresis of specific origin involving his theory of the lowered specific vital energy of the cells and neuron degeneration under the influence of stress, primarily a degeneration of the neuron with secondary changes in the membranes and vessels in consequence of irritation from the products of degeneration.

The paper pointed out and described briefly the somewhat rare manifestations of a pure cerebritic, or of a meningo-cephalitic, pursuing an unexpected and low febrile course of comparatively brief duration; also some of the causes of sudden death in paresis and cerebral syphilis, such as thrombi of the larger arteries, giving illustrative cases. He also spoke of the influence of trauma in hastening or developing paresis and cerebral syphilis and the danger of confounding cause and effect. Several cases were detailed in which accidents, wounds and the like, had been the means by which latent paresis or cerebral syphilis had been brought to notice and pushed forward into rapid development. The effect of previous attacks of simple insanities in paving the way for subsequent paresis was also treated.

DISCUSSION.

Dr. JAMES W. PUTNAM, in the discussion of Dr. Claik's paper, stated that medically it was wrong to treat epilepsy pure, but that the man must be treated. Attention must be given to every physiological function. The patient must be built up. Attention must be given to tissue metabolism, and toxemia from the alimentary tract must be prevented.

As to the high dosage of bromides, it cannot so well be accomplished in private practice as in an institution such as Craig Colony. He urges the use of strontium bromides and advises its use. Other drugs not so beneficial were also alluded to. He further stated that a man with but two attacks of seizures in a year would not go to Craig Colony. This is the class of patients who want to be treated methodically with bromides, and they will not tolerate the large doses of bromides. If bromides don't agree, he uses acetanilid. Prosseau's treatment of atropine or belladonna was also mentioned.

As to the surgical treatment, he takes much the same position as that in the paper, but does not discourage trephining if it shows amelioration of attacks and an increase in the intermissions between the attacks. One should not determine the value of operative procedure by absolute cure, but by an amelioration of the symptoms.

Dr. HERMAN MYNTER agrees with Dr. Putnam that the amelioration of symptoms and length of intermissions between seizures defends the operative measures. He operates, but rarely sees a perfect cure; further, he does not know how many years is to be counted before a case can be said to be free from seizures and therefore cured.

A case was detailed where years after the injury the patient was found in a condition of status epilepticus. An operation was peiformed without the use of an anesthetic and the depressed bone removed. The symptoms disappeared for three years, when again he was called and found the patient in status epilepticus. The patient was operated upon again, more bone being removed and any adhesions which may have formed. After three years he again saw the patient. At this time the seizure was a "petit mal," for which he again operated, since which, so far as is known, the patient is well. Dr. Mynter asked whether this could be considered as a cure. He also mentioned another case operated upon, when the trephined piece of bone showed on its inner surface a spicula of bone which undoubtedly entered the brain substance. It is needless to add that in all his cases he uses the post-operative treatment of bromides.

Dr. Edward J. Meyer submitted a portion of trephined bone, which showed upon the inner surface a spicula of bone which penetrated the brain cortex. It resembles very much the case just mentioned by Dr. Mynter. The case recovered and there has been no attack since the operation. Dr. Meyer also has operated for epilepsy, following the Jannesco method, with a result in the amelioration of symptoms. During a recent visit to Europe he saw Kocher, of Bern, operate for epilepsy, and says that Kocher does not discourage operation on idiopathic cases. In these cases adhesion of the dura are found and gold foil is used. Kocher also drains these cases and overcome cerebral pressure by means of an especially devised silver tube, which Dr. Meyer showed. Kocher drains both sides and even the occipital region.

Dr. Herman G. Matzinger, in discussing Dr. Hurd's paper, first alluded to the Justus test. It is claimed to be of great value in recent and untreated cases of syphilis. A number of cases of primary syphilis sent to him by Dr. Grover Wende gave a positive test, but as this test is dependent on the hemoglobin percentage, and further, as the normal hemoglobin percentage varies from 12 to 20 per cent. according to the Fleischl instrument and other methods, the Justus test cannot be regarded as very reliable.

In regard to syphilis and paresis, Dr. Matzinger mentioned the valuable original work of Straub, of Munich, who found in the ascending and descending arch of the aorta, in 82 out of 89 cases of paresis examined by him, atheromatous changes. He found irregular nodular elevations, which were more marked near the semilunar valves, and the free edges of the leaflets of the valves showed like abnormalities. Similar changes occurring in the vessels of the brain may prove that the phenomena of paresis may be those of starvation.

Dr. ALLEN A. JONES reported a case of epilepsy following a fall down stairs, and asks of the essayist the line of treatment. There was no scar or depression of the head. Is this to be regarded as an idiopathic or traumatic epilepsy? Should bromides be given and are the reflexes, if any, to be corrected?

Dr. WILLIAM C. PHELPS said that in his experience it is a good idea to drain in the operative procedure on an epileptic, and stated a case where, not having the Kocher tube, further not being able to get the tube shown by Dr. Meyer, he used strands of catgut for drainage with a cessation of the convulsive seizures. He believed much improvement can be looked for by using drainage.

Dr. WILLIAM C. KRAUSS stated that the case of bulbar paralysis related by Dr. Mynter was a true bulbar paralysis, but his case was the asthenic form, of which but six cases are recorded in the United States. Referring to the case of status epilepticus briefly reported by Dr. Mynter, where so many operations were required, the patient is now free from epileptic seizures. He referred to a reflex case of epilepsy following an extensive burn, which was treated with potassium bromides with good effect. The Craig Colony at Sonyea is a great boon in the management of epileptics, the regular life, hygiene and healthful occupation is excellent, and compared to it the home treatment, which is a failure.

Dr. Hurd's paper is of great interest from a medico-legal view. He described a legal case where a man was struck and died. His assailant was tried and convicted of manslaughter, receiving fourteen years imprisonment. The autopsy showed a rupture of the basilar artery, which was syphilitic. In his opinion there should be a pardon for the prisoner.

Dr. L. PIERCE CLARK, in conclusion, does not wish it understood that he uses the bromides exclusively; other drugs in individual cases do good. He did not mention the other remedies used in epilepsy, as he desired not to make his paper too long and wished it to conform to the fifteen minute time limit.

Von Bergmann discredits the Kocher method of operation in epilepsy, and many other observers deny an intracranial pressure in idiopathic epilepsy. Kocher's results will be found to result from the breaking up of any possible adhesions and in the relief of intracranial pressure, which necessarily follows operation.

As to Dr. Jones's case, Dr. Clark advises the relief of any possible reflex phenomena and the use of mild sedatives. The traumatic cases of epilepsy should be operated if necessary, but it must be remembered that an operation performed upon any part of the anatomy of an epileptic will often produce diminution or complete cessation of attacks for a year or more. Dr. Clark was at first quite sceptical in the pochlorisation, *i. e.*, reduction of salt in the treatment of epilepsy and began this method of treatment with some reluctance, but is surprised that amelioration has followed the employment of this method. It is best suited to the chronic cases with frequent attacks.

On motion of Dr. Julius Ullman, seconded by Dr. William C. Krauss, a vote of thanks was extended to Dr. L. Pierce Clark for coming to Buffalo and presenting before the Academy his valuable paper.

Members in attendance, 84. Adjourned, 11.30 p. m.

---

### *Section on Ophthalmology, Otology, Rhinology and Laryngology, November 19, 1900.*

Reported by GEORGE F. COTT, M. D., Secretary.

The third meeting of the section of Ophthalmology, Otology, Rhinology and Laryngology for the season was held November 19, 1900.

The chairman being temporarily absent, Dr. J. W. Grosvenor was selected as temporary chairman. Minutes of the previous meeting were then read and approved. During the reading Dr. Frank Whitehill Hinkel arrived and took the chair.

Dr. George F. Cott exhibited a patient, aged 17, from whom he had removed the entire thyroid gland, which was pressing on the trachea laterally, thus causing suffocation. Tracheotomy was first done and a soft catheter passed beyond the portion pressed upon. After twenty-four hours the operation was performed with the assistance of Drs. King, Sage and Bingham. The boy made a good recovery except that he had a partial paralysis of both cords for several weeks.

### DISCUSSION.

Dr. J. W. Grosvenor: Thirty years or more ago Dr. Warren Greene, of Portland, Maine, had performed the operation of thyroidectomy many times, for the arrest of venous hemorrhage. It was his custom to tie the veins with silk ligatures. Whenever a vein bled freely, it was tied as soon as cut. It was the universal belief of the surgeons of his day that the ligature of the veins would almost certainly cause phlebitis or produce some injurious effects upon surgical wounds. Dr. Greene was a pioneer in his method of arresting venous hemorrhage. He was a very successful operator in the extirpation of the thyroid gland.

Dr. HERMAN MYNTER thought it impossible to make a total extirpation of the thyroid gland through a median incision and that the tumor removed must have been a hypertrophied middle lobe. He described the differences between excision of the thyroid and enucleation of other growths in the thyroid gland. The difficulty in excision depended upon finding the proper spot. By an oblique or transverse incision layer after layer is cut till the proper capsule is reached. We reach then only capillary vessels, the gland shells out with ease and little bleeding, while, on the other hand, if we try to excise the gland before reaching the capsule the bleeding from the thin-walled veins may be formidable.

Dr. E. E. BLAAUW asked what the previous treatment had been. He thought that it was a case of parenchymatous degeneration and that early treatment with thyroid extract might have prevented the condition. He had seen quite a number of cases of enlarged thyroids which yielded readily to the extract. One case of special interest was a lady, aged 20, who noticed a swelling about the neck, progressing rapidly during the last six months. She had occasional choking spells. Specific treatment relieved her within two weeks.

In closing, Dr. GEORGE F. COTT said: To Dr. Mynter I would say that the gland was entirely extirpated, as the specimen shows; that it was done through the median incision. In answer to the question of Dr. Blaauw, I know nothing about the previous treatment, as the patient was under the care of another physician and I first saw him on the day tracheotomy was done.

Then followed a discussion on

### LARYNGEAL TUBERCULOSIS.

Dr. DELANCEY ROCHESTER: Laryngeal tuberculosis as a primary disease occurs occasionally, but probably complicates about 30 per cent. of all cases of pulmonary tuberculosis. What causes the laryngeal complication is undetermined. An existing catarrhal laryngitis and the causes determining its occurrence would predispose to the development of tubercular disease in one already suffering from tuberculosis of the lungs. Overuse of the voice, especially under unhygienic conditions, *e. g.* in raw, damp or smoky atmosphere, the abuse of alcohol and the like. But professional vocalists, if they neglect themselves, are more liable to the disease than others. Men are more susceptible than women. The disease usually mainfests itself first in the interarytenoid space, or upon the arytenoid bodies themselves. It may begin anywhere and none of the structure is safe from attack. There is usually a marked pallor of the pharynx and larynx for a period varying from a few days to a week or two before actual lesions show themselves. Finally the changes that occur are swelling of the submucous and mucosa, passing rapidly to ulceration, which may extend to the cartilages, producing perichondritis and chondritis with extensive necrosis and destruction of the parts.

One of the early symptoms of the involvement of the larynx is a feeling of tire in attempting to continue conversation and then inducing cough by talking; then a distinct huskiness, which gradually changes to a true hoarseness, often, finally, to complete loss of voice. The cough is most distressing and often brings on vomiting. If the epiglottis is involved, or the ulceration extends to the pharynx, as it often does, a most distressing dysphagia develops,—distressing from the pain experienced when attempting to swallow and from violent attacks of coughing that are brought on by the attempts to swallow.

Prognostically, there are some cases seen early when there is but little pulmonary involvement, which under judicial local and general treatment recover; but usually the development of laryngeal tuberculosis in a pulmonary case is a bad prognostic factor. I have had cases with infiltration in both lungs and cavities in one recover; but in my own experience I have never known a case of pulmonary tuberculosis develop laryngeal tuberculosis and recover. So, when a case develops hoarseness, I redouble my efforts in treating the laryngeal involvement.

I will take a few minutes to state briefly the steps taken to accomplish this purpose. If the case is on a specific treatment, I increase the dose rather more rapidly than I ordinarily would. I use cleansing laryngeal sprays and insist on continuous inhalations and instruct the patient to cough as little as possible. I also immediately see to it that the nose is thoroughly cleansed daily and put in such a shape that it can perform its proper breathing function. As regards treatment, I will only state that I have had best results with Vaughn's nuclein and creosote. I will leave the subject of local treatment with those who are to follow.

Dr. E. L. Shurly, of Detroit (by invitation): The preceding speaker has touched upon nearly all the salient points of this troublesome and unsettled disease. Treating it from a theoretical standpoint in its relation to general tuberculosis and in regard to its etiology, we might be satisfied by accepting the doctrine of bacilliary origin; yet, as the disease comes under our observation, laryngeal tuberculosis or phthisis brings to the physician many vexatious questions. There is no doubt that primary tuberculosis is probably due to direct infection by tubercle bacilli. But, as clinicians, we must remember that there are certain diseases of this nature characterised by some antecedent degeneration. Therefore, according to the older observers, we may have mixed several diseases under the one name, tuberculosis. On this point, however, there is much difference of opinion. There are conditions both primary and secondary, which must be considered, whether one recognises or not the existence of primary laryngeal tuberculosis.

Statistics regarding laryngeal phthisis are not always reliable. In fact, we have often to "shave them," as bankers say. This is not only true of medical statistics, but of the sciences generally. For instance, we usually harbor three kinds of belief, generally speaking: first, a belief based upon facts; second, upon circumstantial evidence; third, upon pure metaphysical thought, speculation and assumption. An example of belief based upon circumstantial evidence may be drawn from the following case:

A medical friend of mine brought to me a young girl supposed to be suffering with primary laryngeal tuberculosis without involvement of the lungs. The girl was in a good state of nutrition; good family history; had been taking vocal lessons, which the teacher urged her to continue until her throat gave so much trouble that she had to desist. Upon examination, I found a little rise of temperature; no tubercle bacilli in the sputum; moderate swelling of the arytenoids and posterior wall of the larynx; the vocal cords thickened and some enlarged glands in the laryngeal ventricle. The girl seemed to be in good general condition, excepting for the suspiciously frequent pulse. I could find no lesion of lungs by auscultation, percussion and *x*-ray examination; yet she died after a four months course of illness. The post-mortem showed extensive pulmonary infiltration,—an acute general tuberculosis. If it were not for the post-mortem examination in that case, it would no doubt have been always called a case of primary laryngeal tuberculosis, when, as events showed, the lungs were very much involved in the beginning.

It is not common to meet with cases of chronic laryngitis with erosions, and to mistake that condition for tuberculosis. Even though tubercular bacilli may be found, I doubt whether such cases are really tuberculosis. There is only about 13 per cent. of real laryngeal tuberculosis where the lung is involved (pulmonary phthisis), and yet there is every facility in all such cases for infection of the larynx, while really very few become affected. I have repeatedly tried to produce laryngeal tuberculosis in monkeys, but have never yet succeeded, although I have anesthetised the monkey, scarified the laryngeal vestibule and pharynx, and applied either moist or dried sputum. Still such experiments cannot be compared with the conditions surrounding the human being. Therefore, I believe statistics are unreliable and often unfair and valueless for determining the exact origin and nature of laryngeal phthisis.

It is stated that tubercle bacilli have been known to penetrate unbroken mucous membrane. I doubt it! Another clinical aspect is presented in the two types of the disease; for instance, those cases showing hyperemia for a month or more before ulceration, and others showing anemia with or without great swelling. I have myself made the blunder of mistaking a case with great swelling for one of sarcoma or carcinoma. There are all sorts of variation in clinical aspects for which we are not able to account. If it is a fact that bacteria are the sole cause of laryngeal phthisis, we ought to have 90 instead of 13 or 20 per cent. of laryngeal involvement. Alcohol is supposed to be an exciting cause, yet very few chronic alcoholics have the disease. Excessive or bad vocalisation, especially with persons who do not naturally possess a suitable larynx, ought to be a common exciting cause, yet we do not find it so in practice.

In the Michigan forests we formerly met many lumbermen suffering from chronic laryngeal catarrh, yet very few of these cases became laryngeal or pulmonary tuberculosis, although they were constantly exposed to the wind and weather. Regarding treatment, it may be said that some treatment is necessary to neutralise sepsis in all advanced cases. There are cases which show no tubercular bacilli in the sputum at an early stage, but which go right on to general tuberculosis, and others which show the tubercle bacilli early that may go on to cicatrisation. For the local treatment, according to my experience, iodoform is the best agent where any abrasion exists, but this drug avails nothing if no abrasion or ulceration is present. Tannic acid with carbolic acid, tricresol, or the essential oils are useful. Cocaine or morphine used in moderation will relieve odonphagia better than anything.

I have tried tracheotomy in a few cases. One patient was benefited very much, but the others were not. The wounds became very sore from coughing, and there was great inability to expectorate effectually through either the tube or the mouth. I have abandoned this operation altogether in laryngeal phthisis. Regarding inhalations, I believe they do more harm than good in many instances by unduly exercising the parts. The use of some antiseptic which may be evaporated in the bed room at night is often highly beneficial. The real disinfection of the secretions of the larynx and trachea is a questionable accomplishment. Inhalations, however, may be of advantage when properly used in selected cases.

Dr. Shurly, in concluding his remarks, spoke of the advantages of different places for their climatic effects.

Dr. John H. Pryor: Whether tuberculosis of the larynx precedes tuberculosis of the lungs or occurs without any associated pulmonary disease of like character, are questions which have been much discussed in literature. Opinions are divided between clinicians and pathologists. Personally, I am inclined to accept the view of the pathologist that they are practically always associated and that cases in which laryngeal tuberculosis is present without any involvement of the lungs are so rare that we may conclude in almost every instance that the pulmonary lesions are not sufficiently advanced to produce evidence or are overlooked. I have seen cases of tubercular laryngitis in which prominent specialists said that no pulmonary disease was present, but I always found the signs of pulmonary tuberculosis. Often percussion and auscultation are improperly employed and the region of the neck over the apices is slighted. The toxemic symptoms due to laryngeal affection are confusing. Our attention may not be directed to the lung until late because they are ascribed entirely to the infection at one point. Laryngeal tuberculosis occurring early or late with consumption of the lungs is rare in this vicinity,—more rare, in my opinion, than statistics gathered elsewhere would lead us to believe.

An early diagnosis is not such an easy matter as the text-books say. We may have hyperemia and a catarrhal condition as well as the anemia and swelling mentioned. Furthermore, the appearance of anemia and puffiness may be only symptomatic, as the Germans have pointed out, of a general anemic condition characteristic of incipient and pulmonary tuberculosis, as one of the signs of that disease due to toxemia. The disease may be limited to a central portion of the lung and the physical signs delayed. There are reasons for doubting the claim that ulceration of the larynx is principally due to infection from the expectoration passing over a denuded or abraded mucous membrane. We find tubercular laryngitis before there is any elimination of bacilli from the lung, and there is in almost every case of pulmonary tuberculosis catarrhal inflammation, congestion and abrasion of the laryngeal membrane. If this source of infection is as great as has been asserted, certainly the larynx would be affected in a large per cent. of cases.

We are led to the conclusion that there may be other methods of infection, and this may occur through the bloodvessels and lymph channels. This would also account for the involvement of the deeper submucous tissue, which appears sometimes to be the seat of the primary lesion. The most important thing we have learned recently in connection with this subject is the curability of laryngeal tuberculosis. During the last three years I have seen two cases of complete recovery, after a very unfavorable prognosis had been given by men of large reputation and skill. It is occasionally surprising how completely the damage from exudation and ulceration is repaired. Complete aphasia and inability to swallow in the cases mentioned were followed by the entire return of function.

The treatment should not be climatic or local but both; together they promise results which either one cannot accomplish. This fact has been learned in the Adirondacks and at Liberty, Sullivan county, and today one who can afford proper treatment has a better chance. In diagnosticating incipient tuberculosis, either of the larynx or lung, we are prone to forget that the well marked case usually seen is no longer tuberculosis, but a condition of mixed infection and a complex symptom group generally called consumption. True tuberculosis is a very different disease and its diagnosis, treatment and prognosis all depend upon the ability to recognise it. Any discussion of the local treatment of laryngeal tuberculosis seems to me to be beyond the range of the topic under consideration.

Dr. CHARLES CARY said his main object in speaking was to put in a good word for the larynx, which certainly is the great friend of all physicians and the mainstay for some. He thought the vulnerability of the larynx to tuberculosis was but slight when one considers the degree to which it is exposed to infection both from without and from "bathing" from within in pulmonary tuberculosis. His views fully coincided with those of Dr. Pryor, that it was a late manifestation in advanced phthisis, that it was not as common, in his experience, as authorities would lead him to expect, that it was curable only in those cases where the pulmonary symptoms subsided; but that he could not believe that Dr. Pryor's views were correct when he stated that laryngeal affection took place through the lymphatics as bloodchannels, as the infection would have to float against the stream to reach the larynx from the lungs.

Dr. Cary then gave a brief description of the pulmonary and laryngeal lymphatic chain to substantiate his statement, and closed by calling in question the validity of Dr. Shurly's experiment on monkeys when he insufflated the tubercular virus on excoriated surfaces. He said he did not know the resistance that the tubercle bacillus had to ether or chloroform vapor, but he thought the monkeys under ether would receive only attenuated virus if, as Dr. Shurly said, these experiments had been carried on under an anesthetic.

Dr. E. E. Blaauw.—I wish to state that I do not agree with Dr. Cary. We must accept a propagation of the disease against the lymph current. We see too often a one-sided tuberculous affection in the larynx corresponding to the localisation of the tubercular process in the lung on the same side, and when the last one is that which is primarily affected. Now, this is not so remarkable because we often find in carcinoma of the liver or stomach supraclavicular involvement of the lymphatic glands. In carcinoma of the breast, the involvement of the glands of the armpit can only be explained by the fact that the disease has beeen propagated against the lymph current.

In tuberculous cases we should not only look for anemia of the pharynx and larynx, as the previous speaker intimated, but also at the hard palate, a locality open to every observer. The high percentage of tuberculous larynges that the pathologists find must be explained in part by the fact that all abnormalities found on the post-mortem table are called tuberculous, while many of them developed only in the last days of life and others are not specific whatever.

As enlarged tonsils and adenoids may be a portal of entry, we should remove them, which will go a great way toward prevention, because proper inhalation through the nose will then be obtained, and one of the many disadvantages of mouth-breathing cut short. Another not insignificant one is improper development of the chest and therefore predisposition toward the development of tuberculosis pulmonum. Here the duty of the family physician shows itself clearly. Fellows present, 56.

---

*Section on Obstetrics and Gynecology, November 27, 1900.*

Reported by WM. G. RING, M. D., Secretary.

The regular monthly meeting of this section was held at the Academy rooms Tuesday evening, November 27, 1900.

The meeting was called to order at 8.45 p.m. by the chairman, Dr. A. J. Colton.

The minutes of the previous meeting were read and approved.

Dr. Herman E. Hayd reported four cases of abdominal hysterectomy, operated on by him during the past week, giving the details of each case and showing the specimens.

Dr. Wm. R. Stone, of New York City, read a paper entitled, Cocainisation of the Spinal Cord by Means of Lumbar Puncture in Labor.

European physicians in giving the details of the operation of lumbar puncture entirely overlook the American literature on the subject. Dr. Corning discovered the method and published his ideas over fifteen years ago. Medullary anesthesia lasts from thirty minutes to four and one half hours; uterine contractions are not interfered with.

The skin area in which lumbar puncture is to be made should be thoroughly cleansed and disinfected. The syringe should be of the solid piston type; the needle should be 9 c.m. long and of short beveled point. The puncture is made between the third and fourth lumbar vertebra, from below upward, the patient being in the so-called scorchers position, or in an exaggerated Sims's position.

When the needle is in spinal canal withdraw a few drops of the spinal fluid in order to be certain that the canal has been penetrated. Use ten minims of a 2 per cent. solution of cocaine, or 1-6 of a grain solution, to be in a two dram vial and boiled therein in boiling water for ten minutes. This method does not change cocaine, chemists to the contrary notwithstanding. A spray of ethyl chloride may be used at point of puncture to anesthetise the skin.

Dangers: (1) Introduction of pathogenic germs; (2) Personal idiosyncrasy of the patient to the effects of cocaine, which may be avoided by repeated small injections; (3) Piercing spinal cord; this is usually not followed by bad results. Wait until cervix uteri is thin and dilatable and pain in back is severe. Anesthesia takes place in from two to twelve minutes.

Effects of injection: Spasm in the parts is done away with. Tingling and coldness in lower extremities; vomiting; numbing of pains to a mere pin prick; in fifteen minutes patient is pain free.

The headache which usually follows three hours after the injection may be controlled by hypodermic injection of morphine, or 1-50 of a grain of glonoin. Hyoscine hydrobromate, 1-150–1-200 grain, given hypodermatically before puncture is made prevents both vomiting and headache.

After delivery the temperature rises from 101 to 103° Fahrenheit; after sixteen hours it falls to normal. Pulse is little accelerated. Experiments with other drugs give the same results minus anesthesia.

DISCUSSION.

Dr. Marcel Hartwig mentioned two cases in Buffalo in which this method was used. He then reported two cases he had seen in New York in the practice of Dr. Grandin, Dr. Marx making the injections. One was a signal failure; the patient felt everything; she felt pain from needle in skin although kelene was used as a local anesthetic at point of puncture; the pain deeper was acute. After escape of spinal fluid he injected 1-5 grain of cocaine and waited; no anesthesia. Cyanosis with vomiting supervened, the pulse rate being between 120--130 per minute. Dr. Grandin made an abdominal incision, the patient shrieking with pain; it was not ideal surgery, to say the least.

The second case was one of pelvic abscess; the operation, vaginal section; the result was similar to the first—the patient made such a noise that the next patient refused to be operated on. Dr. Hartwig then gave extracts from cases reported in various journals; all unfavorable except the last, a case reported from Chicago. In concluding, Dr. Hartwig said we must find the status of both sides of the question.

Dr. A. L. Benedict asked the essayist this question: How far up is the edge of the upper segment of anesthesia?

Dr. Sydney Dunham objected to the cases cited by Dr. Hartwig, on the grounds that they were not cases of labor. There is the same difference there is in other cases. He then gave the details of two labor cases seen in the service of Dr. Stone at the City Hospital, New York. There were advantages in the use of this method; chloroform predisposes to hemorrhage; ether slows labor pains; it saves the expense of the service of an anesthetiser. The bevel of the needle should be short.

Dr. Herman E. Hayd thanked Dr. Stone for bringing the subject here. Perhaps the reason it had not been used more here was on account of the conflicting reports seen by all of us. Dr. Grandin said he would not use the method again until ten thousand cases had been reported. Would not accept this method without the reservations Dr. Stone advocates. The general practitioner should not dabble around the spinal cord with this method. I am going to try this method at once.

Dr. LUDWIG SCHROETER discussed the paper from the standpoint of reported cases and advocated the use of chloroform as a safe anesthetic in labor, and does not advocate medullary anesthesia in normal labor, but does where os uteri is rigid, in ruptured perineum and retained placenta, or in any operation performed after labor has terminated, and in breech presentations with dorsum posterior, transverse cases. He does not believe it will take the place of chloroform in operative cases. The future will show how much we can trust it.

Dr. STEPHEN Y. HOWELL described the modus operandi of the production of the bad effects of cocaine in spinal puncture. Passing by diffusion through the foramen of Le Gendre into the fourth ventricle of the brain where are located the vomiting center, the cardiac center, the very foundation of the structures of life are attacked.

Dr. WM. STONE, in closing the discussion said that he had personally known those cases spoken of by Dr. Hartwig. Dr. Marx had only introduced the needle three times before using it on Dr. Grandin's cases, and gave the reasons why the method might fail to yield results. The zone of anesthesia is only limited upward by the extent of the diffusion of the injection. He does not expect to see the method become universal. For diagnosis of cerebro-spinal fever, lumbar puncture is good. The method is not indicated in every case of labor. Since the technique of the operation has become more perfect the results have been better. The procedure itself is harmless.

Dr. DUNHAM moved a vote of thanks to Dr. Stone.

Carried.

Dr. EUGENE A. SMITH read a paper entitled, Appendicitis, Complicating Labor; report of a case.

Dr. HERMAN E. HAYD in discussing Dr. Smith's paper said that he believed that many of the aches and pains complained of were due to this condition; he believed that many cases reported were not appendicitis. Another reason why we do not get appendicitis in pregnant women: during pregnancy all the pelvic vessels are distended and filled with blood and naturally the little vessels to the appendix, which exists in women from the ovarian artery, makes a richer supply of blood to the appendix.

Number present, 70.

Meeting adjourned at 10.45 p.m.

